# Oral squamous cell carcinoma detection using EfficientNet on histopathological images

**DOI:** 10.3389/fmed.2023.1349336

**Published:** 2024-01-29

**Authors:** Eid Albalawi, Arastu Thakur, Mahesh Thyluru Ramakrishna, Surbhi Bhatia Khan, Suresh SankaraNarayanan, Badar Almarri, Theyazn Hassn Hadi

**Affiliations:** ^1^Department of Computer Science, College of Computer Science and Information Technology, King Faisal University, Al-Ahsa, Saudi Arabia; ^2^Department of Computer Science and Engineering, Faculty of Engineering and Technology, JAIN (Deemed-to-be University), Bangalore, India; ^3^Department of Data Science, School of Science, Engineering and Environment, University of Salford, Salford, United Kingdom; ^4^Department of Electrical and Computer Engineering, Lebanese American University, Byblos, Lebanon; ^5^Applied College in Abqaiq, King Faisal University, Al-Ahsa, Saudi Arabia

**Keywords:** Oral Squamous Cell Carcinoma, histopathological images, diagnostic precision, microscopic imaging, cancer identification, EfficientNet

## Abstract

**Introduction:**

Oral Squamous Cell Carcinoma (OSCC) poses a significant challenge in oncology due to the absence of precise diagnostic tools, leading to delays in identifying the condition. Current diagnostic methods for OSCC have limitations in accuracy and efficiency, highlighting the need for more reliable approaches. This study aims to explore the discriminative potential of histopathological images of oral epithelium and OSCC. By utilizing a database containing 1224 images from 230 patients, captured at varying magnifications and publicly available, a customized deep learning model based on EfficientNetB3 was developed. The model’s objective was to differentiate between normal epithelium and OSCC tissues by employing advanced techniques such as data augmentation, regularization, and optimization.

**Methods:**

The research utilized a histopathological imaging database for Oral Cancer analysis, incorporating 1224 images from 230 patients. These images, taken at various magnifications, formed the basis for training a specialized deep learning model built upon the EfficientNetB3 architecture. The model underwent training to distinguish between normal epithelium and OSCC tissues, employing sophisticated methodologies including data augmentation, regularization techniques, and optimization strategies.

**Results:**

The customized deep learning model achieved significant success, showcasing a remarkable 99% accuracy when tested on the dataset. This high accuracy underscores the model’s efficacy in effectively discerning between normal epithelium and OSCC tissues. Furthermore, the model exhibited impressive precision, recall, and F1-score metrics, reinforcing its potential as a robust diagnostic tool for OSCC.

**Discussion:**

This research demonstrates the promising potential of employing deep learning models to address the diagnostic challenges associated with OSCC. The model’s ability to achieve a 99% accuracy rate on the test dataset signifies a considerable leap forward in earlier and more accurate detection of OSCC. Leveraging advanced techniques in machine learning, such as data augmentation and optimization, has shown promising results in improving patient outcomes through timely and precise identification of OSCC.

## 1 Introduction

Oral Squamous Cell Carcinoma (OSCC) stands as one of the most prevalent malignancies originating from the epithelial cells within the oral region.

### 1.1 OSCC

Oral Squamous Cell Carcinoma (OSCC) holds a significant position among the various malignancies affecting the epithelial cells in the oral cavity. Its prevalence globally contributes substantially to the overall burden of cancer-related health issues, resulting in significant morbidity and mortality rates. The intricate development of OSCC involves a multifaceted interplay of ecological factors, including environmental influences and lifestyle choices. The utilization of betel nuts, tobacco chewing, alcohol consumption, human papillomavirus (HPV) infection, and poor oral hygiene significantly escalates the susceptibility to developing OSCC.

This particular form of cancer primarily manifests as localized lesions within distinct regions of the oral cavity, encompassing areas such as the lips, tongue, lower mouth region, palate, gingiva, and buccal mucosa. In its initial stages, OSCC may exhibit subtle indications, such as persistent ulcers or the presence of white or red patches known as leukoplakia or erythroplakia, respectively. These seemingly innocuous signs can progress into larger lesions, leading to symptoms like pain, difficulty in swallowing, or impaired speech.

The prevalence and severity of OSCC are closely associated with a variety of risk factors. Betel nut usage, a common practice in several regions, significantly heightens the risk of developing OSCC. The habitual chewing of tobacco, in various forms, has also been strongly linked to the incidence of oral cancer, including OSCC. Additionally, the consumption of alcohol, particularly in excessive amounts over prolonged periods, serves as another notable contributor to the development of this malignancy.

Human papillomavirus (HPV) infection, specifically certain high-risk strains, has emerged as a significant risk factor for OSCC, particularly in certain subsets of the population. Its presence in the oral cavity can augment the likelihood of developing this form of cancer. Moreover, poor oral hygiene practices, which encompass inadequate dental care and hygiene routines, can further compound the risk factors associated with OSCC.

Clinically, the presentation of OSCC varies but often showcases itself through localized lesions within the oral cavity. These lesions can arise in diverse areas, including the lips, tongue, lower mouth region, palate, gingiva, and buccal mucosa. In its early stages, OSCC may demonstrate subtle symptoms, such as persistent ulcers or the presence of white or red patches (leukoplakia or erythroplakia). These seemingly benign indications can progress into larger, more conspicuous lesions that lead to discomfort, difficulty in swallowing, or impaired speech. The diagnosis of OSCC typically involves a comprehensive examination, including tissue biopsies, imaging studies, and other relevant tests to confirm the presence and extent of the malignancy.

Treatment strategies for OSCC often encompass a multidisciplinary approach, combining surgical interventions, radiation therapy, and chemotherapy, depending on the stage and extent of the disease. Early detection and intervention significantly enhance the prospects of successful treatment outcomes and improved survival rates. Additionally, lifestyle modifications, cessation of high-risk behaviors like tobacco chewing and excessive alcohol consumption, and the implementation of proper oral hygiene practices play pivotal roles in preventing the onset and progression of OSCC.

### 1.2 Diagnostic significance of histopathology

Histopathological examination of tissue samples remains the cornerstone for OSCC diagnosis. This process involves the microscopic analysis of tissue biopsies obtained from suspicious lesions within the oral cavity. Pathologists meticulously scrutinize cellular morphology, tissue architecture, and nuclear features, identifying malignant changes characteristic of OSCC.

### 1.3 Challenges in diagnosis

The diagnosis of OSCC through histopathology demands an expert pathologist since it is highly complex and variable in cellular presentations. Distinguishing between benign conditions, dysplasia, and invasive carcinoma requires meticulous examination and may sometimes pose diagnostic challenges, leading to the need for multiple biopsies or ancillary tests. Oral Squamous Cell Carcinoma (OSCC) remains a formidable challenge in the field of oncology, impacting both public health and affected individuals profoundly. Its prevalence, late-stage detection, diagnostic intricacies, and comprehensive impact necessitate concerted efforts in prevention, early detection, and advanced treatment modalities to alleviate the burden it poses on individuals and healthcare systems.

### 1.4 Significance of automated detection in medical imaging

The rise of deep neural networks and their application in medical imaging has revolutionized disease detection and diagnosis. Automated detection systems utilizing deep learning algorithms demonstrate remarkable potential in analyzing medical images with efficiency, accuracy, and speed. In the context of OSCC, these systems offer the prospect of streamlining the diagnostic process, enabling early detection, and improving patient outcomes. Automated analysis of histopathological images can aid in precise identification and classification of cancerous tissues, augmenting the capabilities of pathologists and reducing diagnostic turnaround times.

### 1.5 Motivations

#### 1.5.1 Context establishment

Oral Squamous Cell Carcinoma (OSCC) represents a significant global health burden, accounting for a substantial portion of oral malignancies. Despite advancements in oncology, early detection of OSCC remains a critical challenge. The absence of precise diagnostic tools hampers the timely and accurate identification of this condition, often leading to delayed diagnoses and subsequent implications for patient outcomes.

#### 1.5.2 Clinical importance

The impact of early OSCC detection on patient prognosis cannot be overstated. Timely identification facilitates earlier intervention, potentially enhancing treatment efficacy and overall survival rates. OSCC, when detected at advanced stages, presents significant challenges in treatment modalities and may result in more invasive therapies with reduced success rates. Therefore, establishing precise and reliable diagnostic methodologies holds paramount importance in improving patient care and outcomes.

#### 1.5.3 Technological gap

Existing diagnostic methodologies for OSCC exhibit limitations in accuracy, efficiency, and discernment between normal epithelium and cancerous tissues. The current landscape lacks tools that can reliably differentiate between these tissue types in histopathological images, leading to diagnostic ambiguities and subsequent challenges in providing effective treatment strategies.

The outcomes of this proposed study are summarized as follows:

- Development of a tailored deep neural network model, built on EfficientNetB3, integrating cutting-edge methods (data augmentation, regularization, optimization) to differentiate normal oral tissue from Oral Squamous Cell Carcinoma (OSCC) samples.
- Emphasis on the use of deep learning to tackle OSCC diagnostic hurdles, potentially enhancing early and precise detection, ultimately enhancing patient prognosis.
- Validation of the model’s reliability and its potential utility in oncology through the utilization of a publicly accessible dataset, showcasing clinical application viability.

The remaining part of the paper is structured as: Section 2 provides a review of the related works. Section 3 introduces the proposed methodology. Section 4 provides the results and discussions along with the comparison to state-of-art existing works and section 5 concludes the study with future directions.

## 2 Related work

oral Squamous Cell Carcinoma (OSCC) diagnosis research has boomed in recent times, driven by the urgent need to detect and identify this common oral cancer early and accurately. However, in any scientific field, there are limitations to existing approaches, creating opportunities for further investigation and innovation. The primary aim of our research is to create a new system which improves the accuracy of current datasets and analyses them more efficiently and identify the areas for future research study and improvement.

In the early paper (1), the author developed a deep learning framework for the automatic detection and categorization of oral cancerous cells in histopathology images. The framework was 92% accurate in identifying oral lesions and 90% accurate in classifying oral lesions as OSCC or non-OSCC. This is more accurate than human specialists. In (2), the author introduced a novel deep learning framework for OSCC diagnosis through transfer learning where they develop a deep learning framework for OSCC diagnosis using transfer learning. The accuracy rate was 93%, placing it on par with the diagnostic capabilities of human pathologists. (3), in the paper, presented an innovative approach where author propose a framework based on the realm of deep learning consisting of an in between layer for diagnosing OSCC from histopathological images where it achieved an accuracy of 95%. Most of the researchers worked on histopathological imaging database for oral cancer analysis (HID-OSCC).

Rahman et al. (4) employed a technique using Gray-Level Co-occurrence Matrix (GLCM) along with histogram which was used for feature extraction. In this test statistical analysis methods such as *t*-test with principal component analysis were used to extract out the featured. In this approach they were able to achieve a significant accuracy of 89.7% (4).

Fu et al. (5) utilized a cascaded Convolutional Neural Network (CNN) for OSCC detection from photographic images. Multiple hospital-derived images underwent augmentation through image processing. Their model, evaluated using Transfer Learning and Receiver Operating Characteristic (ROC) curves, achieved an exceptional Area Under the Curve (AUC) of 0.995, along with 95.3% accuracy, 97.4% sensitivity, and 93.5% specificity (5).

Rahman et al. (6) used multiple classifiers in another research resulting in better accuracy and results. In all these papers multiple fusion techniques were used and all of them were significant for their contribution which included there optimal accuracy gain along with their percepts of addition to the existing technologies.

During our literature work only, we found that MobileNet for CNN can be used as one of the better solutions which results in a good sensitivity with varying images (7).

According to the study, *ex vivo* fluorescence confocal microscopy data analysis can be used to diagnose oral squamous cell cancer (OSCC). With many advantages and some drawbacks remaining, the study pointed out the necessity for more analysis to create deep learning models that are more reliable and understandable for this imaging modality, nevertheless. To advance accurate and reliable OSCC diagnosis using deep learning approaches, it is crucial to address difficulties relating to model interpretability, data quality, and intermodal variability.

Table 1 provides a comparative analysis of various studies focusing on Oral Squamous Cell Carcinoma (OSCC) detection techniques along with their respective datasets and achieved accuracies.

**Table 1:** Related works in the field.

Research study	Dataset	Technique	Remarks
Ananthakrishnan et al. (2023) (8)	HID-OSCC	The use pretrained CNN and Bounding box for the oscc classification.	In this technique they achieved an accuracy of 96.94%
Fatapour et al. (2023) (9)	SEER database	Gradient Boosting Machine Model for Detecting the recurrence.	Through the technique they achieved an accuracy of 81.8%
Das et al. (2023) (10)	HID-OSCC	Fusion of multiple techniques and pretrained models including Resnet50, ResNet101, Vgg19 and Mobile Net.	The fusion resulted in accuracy of 97.82%
Nagarajan et al. (2023) (11)	Multiple datasets	MobilenetV3 with Gorilla Troops Optimizer	The optimizer enhanced the accuracy up to 95%
Flügge et al. (2023) (12)	Private dataset	Swin-Transformer	The swin transformer with CNN enhanced accuracy at 98.6%
Haq et al. (2023) (13)	HID-OSCC	Filtering technique such as Gabor with its fusion to ReNet50 along with CatBoost classification.	Filtering resulted with an accuracy of 94.92%
Deif et al. (2022) (14)	Private dataset	Inception V3 with BPSO for optimization and classification.	Optimization reduced computational cost and enhanced accuracy upto 96.3%
Rahman et al. (2022) (2)	Histopathological imaging database for Oral Cancer analysis	Use of alexnet for better classification.	Alexnet as classifier did it job well and resulted in accuracy of 90.06%
Alanazi et al. (2022) (15)	Public datasets	IDL-OSCDC model for improvised Deep Learning Feature Extraction.	The technique enhanced accuracy up to 95%
Wu et al. (2022) (16)	Public datasets	TMA annotated images for CNN.	The annotated images resulted in accuracy of 95.8%

This comparative assessment serves as a reference point to understand the nuanced attributes and considerations associated with both the gorilla-inspired optimization and CNN-based algorithms in the context of our research objectives.

## 3 Methodology

**Figure 1:**
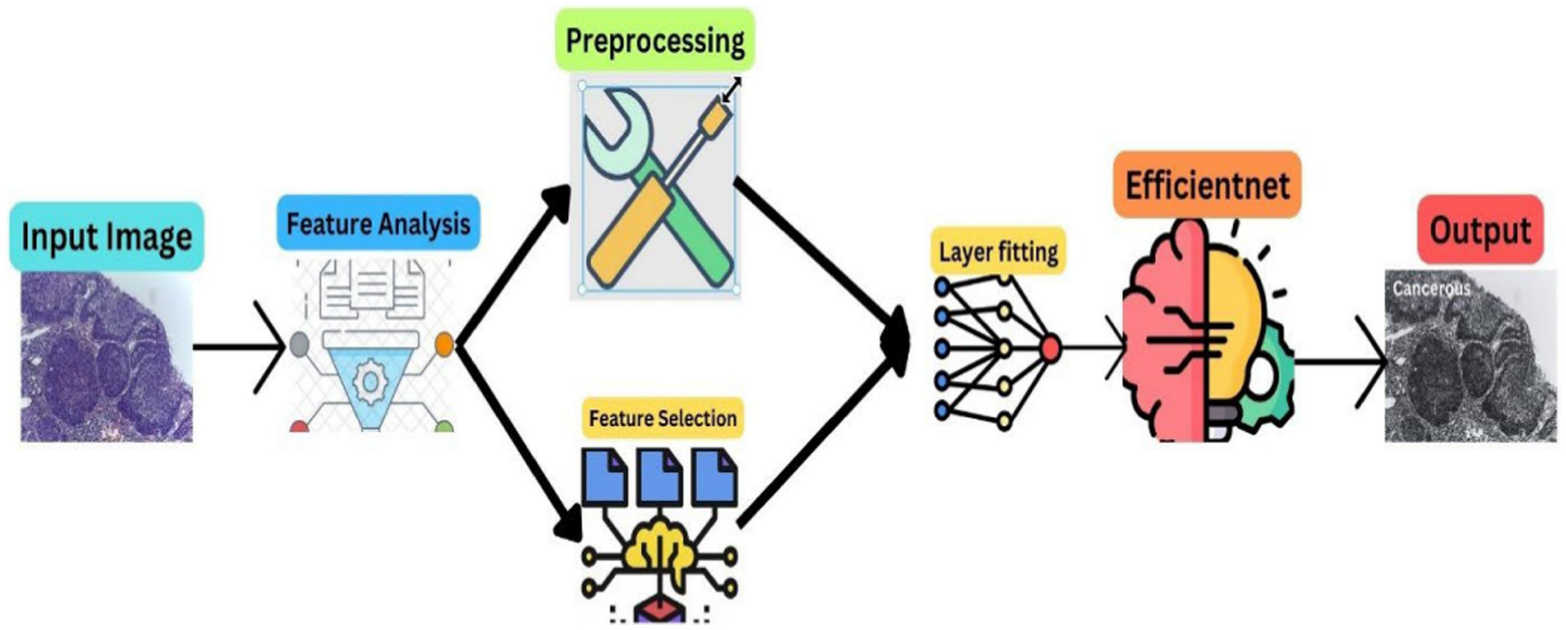
Model architecture.

The methodology of this research is anchored in the advanced realm of deep learning, particularly focusing on the analysis of a comprehensive dataset of histopathological images. These images, pivotal in medical diagnostics, are instrumental for the accurate identification and classification of normal epithelial cells and oral cancerous cells within the oral lesions. The dataset, an extensive collection of 1,224 images, is meticulously categorized into two distinct sets differentiated by their resolution. This categorization is not arbitrary; it is a deliberate attempt to distinctly highlight the variations between normal epithelial tissues and OSCC manifestations. In our proposed methodology, we used the robust computational power of Convolutional Neural Networks (CNNs), with a special emphasis on the EfficientNetB3 architecture, to realize a dual objective: achieving high accuracy and ensuring efficiency in the classification of the dataset. Figure 1 depicts the proposed model workflow.

### 3.1 Data collection and preparation

#### 3.1.1 Data sources

**Figure 2:**
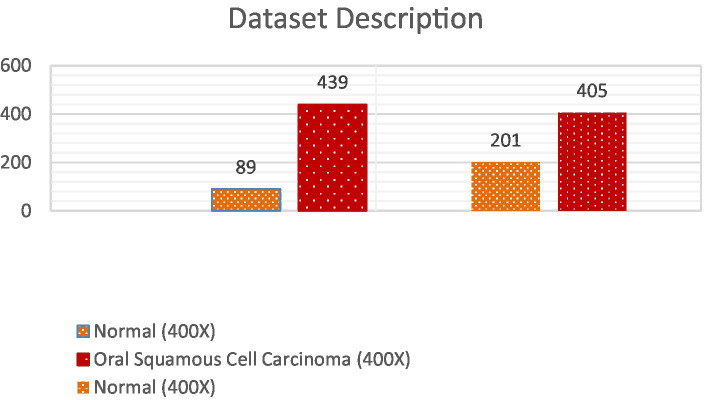
Dataset distribution.

The dataset employed in this investigation comprises 1,224 publicly accessible images. These images are segregated into two distinct collections, each exhibiting varying resolutions. The initial collection encompasses 89 images displaying normal epithelial tissue of the oral cavity and 439 images depicting Oral Squamous Cell Carcinoma (OSCC) at a magnification level of 100x. Meanwhile, the secondary collection encompasses 201 images exhibiting normal oral epithelium and 495 histopathological representations of OSCC at a magnification of 400x. These images were captured via a Leica ICC50 HD microscope, utilizing H&E staining on tissue slides that were meticulously assembled, processed, and classified by proficient medical specialists, sourced from 230 individual patients (17). Image data distribution is shown in Table 2 and data description in Figure 2.

**Table 2:** Image data distribution.

100X magnification	
Normal (100X)	89
Oral Squamous Cell Carcinoma (100X)	439
400X magnification	
Normal (400X)	201
Oral Squamous Cell Carcinoma (400X)	405

#### 3.1.2 Data organization

**Figure 3:**
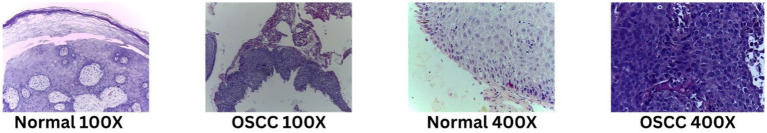
Sample images from dataset under different magnification.

The organization of the dataset plays a vital role in the overall efficiency of the processing phase. To facilitate ease of access and processing, the images are meticulously sorted into separate directories based on their categorical classification – normal epithelium or OSCC. The structured arrangement is not solely for convenience; it stands as a strategic choice profoundly simplifying data management and labeling during the critical preprocessing phase. This approach sets the foundation for enhanced accuracy in subsequent analysis. Figure 3 shows some sample images.

#### 3.1.3 Data processing

**Figure 4:**
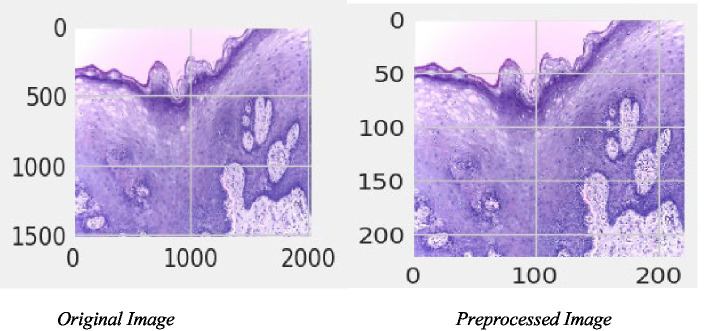
Original and preprocessed image.

Processing the image data in this study involves several well-defined steps. The initial phase involves the careful loading of images from their respective directories. Considering the high-resolution nature of these images, they are resized to a consistent dimension, a step that is essential for maintaining uniformity across the dataset and ensuring computational efficiency. Furthermore, the study employs various image augmentation techniques, such as horizontal flipping. This is not just a mechanical step but a strategic one, aimed at enriching the dataset and enabling the model to learn from a more diverse set of patterns and features, thereby enhancing its ability to accurately classify and differentiate between various cell types. Figure 4 depicts the original and pre-processed image.

### 3.2 Detailed data analysis (DDA)

#### 3.2.1 Data visualization

In this finding, the use of seaborn and matplotlib libraries is not just a technical requirement but a strategic tool in our data analysis arsenal. These libraries are employed to craft various types of plots, including bar charts and pie charts, each serving a unique purpose in representing the distribution of data across different categories. The visualizations produced are not mere representations of data; they offer deep insights into the balance and composition of the dataset, and critically, they help in identifying any potential biases or irregularities that could skew the study’s findings. This visual approach to data analysis is a powerful method to ensure the integrity and reliability of the research.

- Let *D* represent the dataset.
- *P* the plotting function
- *V* the visualization *I* the insights generated.

The process of using seaborn and matplotlib libraries for data visualization can be represented symbolically as in equation 1.

- Furthermore, insights (*I*) are derived from the visualization (*V*) using a function *F*:

(1) I=F(V)

#### 3.2.2 Missing value analysis

The integrity of the dataset is paramount in this study. As such, a key focus area is the identification and handling of missing data. To achieve this, we employ sophisticated techniques like matrix visualization for detection of missing value. Addressing missing values is not a universal fix; it’s a meticulously planned procedure. Strategies like data imputation or removal are selectively utilized based on the specific characteristics and magnitude of the absent data, ensuring a tailored approach to handling these gaps. This meticulous approach to handling missing data is crucial in maintaining the overall integrity and quality of the dataset, which in turn, is pivotal for the accuracy of the study’s outcomes.

- Let *D* represent the dataset.
- *M* the missing values
- *H* the handling strategy.
- Now the working mechanism can be seen in equations 2, 3.

(2) M=Identify(D)

(3) H=Handle(D,M)

### 3.3 Data preprocessing for deep learning

#### 3.3.1 Data splitting

**Figure 5:**
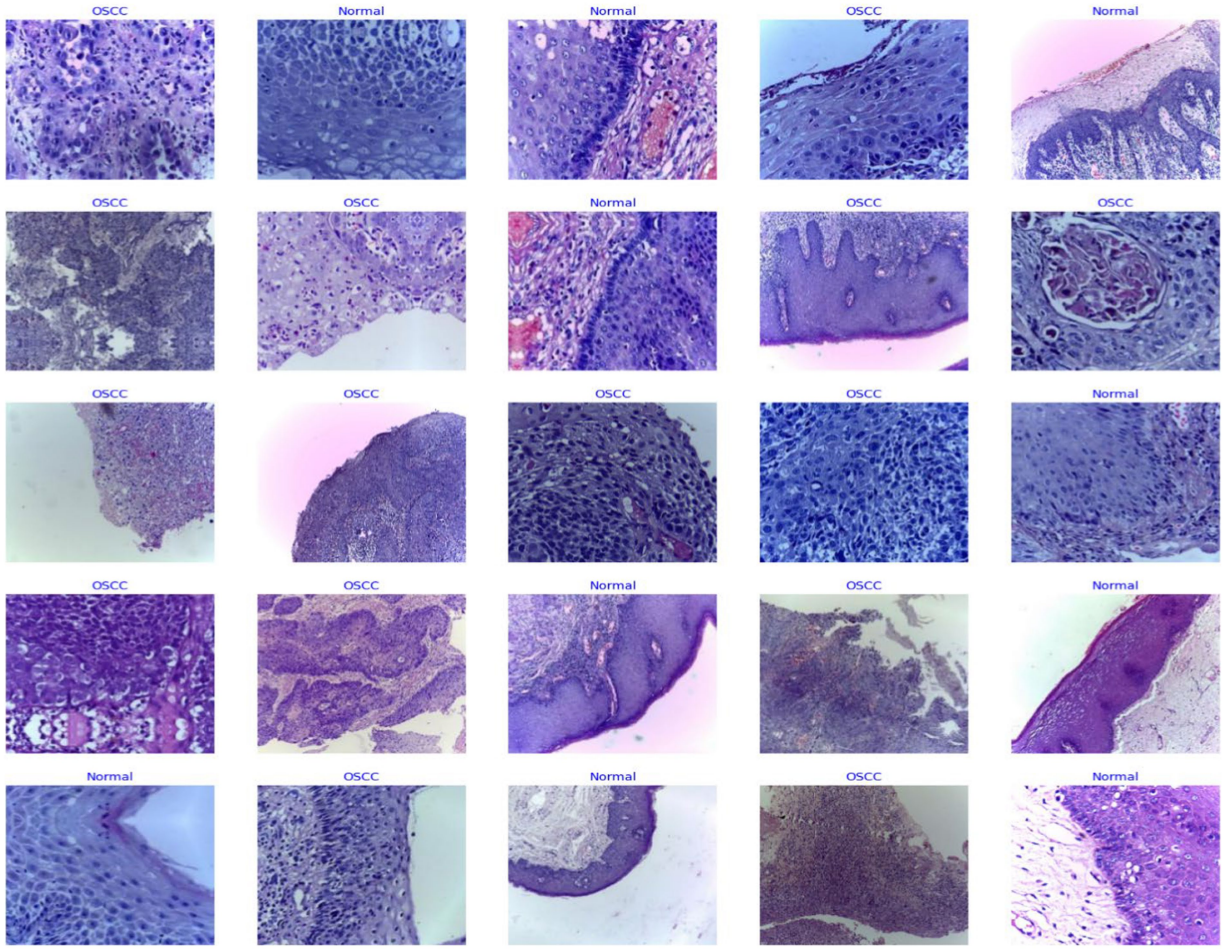
Annotated images with labels.

Within the domain of deep learning, how a dataset is partitioned holds substantial sway over the model’s performance and its capacity to generalize to novel data. In this study, a deliberate division of the dataset occurs, segregating it into three distinct subsets: training, validation, and testing, ensuring a strategic approach to model development and evaluation. Figure 5 shows the annotated images.

**Training Set**: This subset, the largest of the three, is the main driver of the model’s learning process. It provides a diverse array of examples from which the model can learn the distinguishing features of normal epithelial cells and OSCC.

**Validation Set**: This set acts as a checkpoint during the model training process. It is not involved in the actual training but is employed periodically to evaluate the model’s computational performance. This helps in fine-tuning the model’s parameters and provides an early indication of overfitting.

**Testing Set**: The ultimate evaluation of the model’s performance occurs within this subset, encompassing completely unfamiliar data. This segment provides an authentic gauge of the model’s ability to generalize and effectively perform in real-world scenarios, avoiding biases from prior exposure to the model during training or validation phases.

- D represents the entire dataset.
- T symbolizes the training set.
- V denotes the validation set.
- S represents the testing set.

So, the splitting of the dataset can be seen in equation 4.


(4) T,V,S=Split(D)

The splitting of the dataset can be represented by equation 5:


(5) D=T∪V∪S

#### 3.3.2 ImageDataGenerator

Keras’s ImageDataGenerator is a cornerstone in the preprocessing phase for a few pivotal reasons:

- **Real-time Data Augmentation**: This feature allows the expansion of the dataset by generating altered versions of the images, such as rotated or zoomed-in images. This augmentation helps in building a model that is robust and less prone to overfitting and is elaborated in equation 6.
- **Tensor Conversion**: It converts image files into tensors, which are the required input format for training neural network models which is gained using equation 7.
- **Parameter Tuning**: Parameters like rescaling, zoom range, and horizontal flip are carefully selected to enhance the dataset without distorting the essential features of the images as can be seen in equation 8.
- Let *I* represent the original image dataset.
- *A* the augmented dataset.
- *T* the tensor format dataset.
- *P* the parameter set.

(6) A=Augment(I,P)

(7) T=ConvertToTensor(A)

(8) P=TuneParameters()

**Figure 6:**
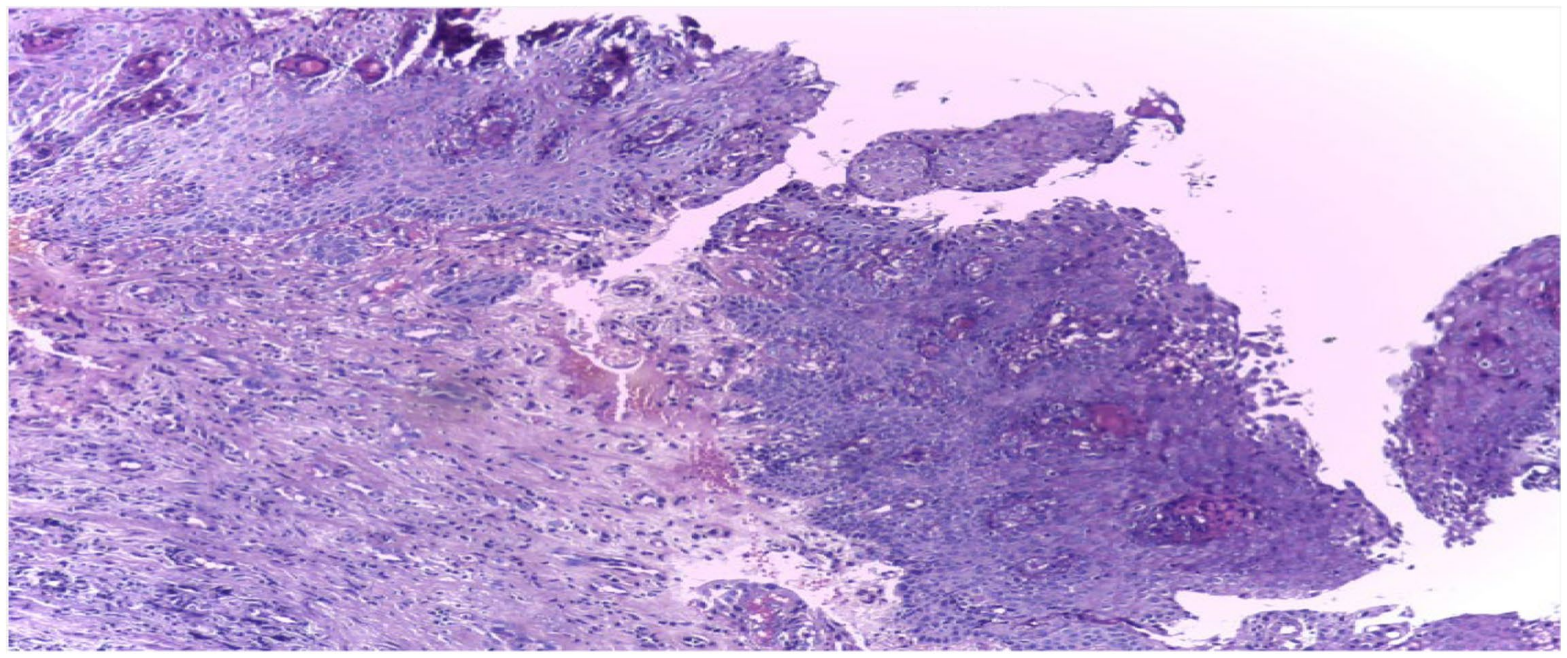
Augmented image.

The augmented image is shown in Figure 6.

### 3.4 Deep learning model development

#### 3.4.1 Model architecture

The pivotal element influencing the success of this research is the model architecture. The selected architecture is EfficientNetB3, a member of the EfficientNet series acknowledged for its efficacy and superior accuracy in image classification endeavors. This model encompasses:

1. **Convolutional Layers**: These layers are fundamental in extracting features from the images. They use filters to capture patterns such as edges, textures, and other relevant details. Output of the convolution layers is computed as shown in equation (9).

(9) $$O_{C}=\mathrm{Conv}\left(I,F_{C}\right)$$

Where,

- *I*: Input to the convolutional layers
- *F_C_*: Convolutional filters
- *O_C_*: Output of the convolutional layers

1. **Pooling Layers**: After convolutional layers process the input, pooling layers play a role in diminishing the spatial dimensions (height and width) of the resultant volume. This reduction facilitates subsequent convolutional layers in their processing of the data. This reduction is crucial for decreasing the computational load and the number of parameters. It is shown in equation (10).

(10) $$O_{P}=\mathrm{MaxPool}\left(I_{P},S\right)$$

Where,

- *I_P_*: Input to the pooling layers
- *S*: Pooling size

1. **Fully Connected Layers**: These layers, positioned toward the end of the network, perform high-level reasoning in the neural network and are essential for the classification task. Output of the fully connected layers is shown in equation (11).

(11) $$O_{F}=\mathrm{Activation}\left(I_{F}\times W+B\right)$$

Where,

- *IF*: Input to the fully connected layers
- *W*: Weights
- *B*: Biases
- *O_F_*: Output of the fully connected layers

#### 3.4.2 Pre-trained models

EfficientNetB3, part of the EfficientNet family, is selected for its unique scaling method that balances network depth, width, and resolution, which contributes to improved accuracy and efficiency. The model has undergone pre-training on the extensive ImageNet dataset, renowned for its vast array of diverse image categories. This pre-training provides the model with foundational knowledge and feature extraction capabilities across a wide spectrum of visual information.

This pre-training endows the model with a rich feature-detection capability, significantly enhancing its performance on the histopathological image dataset.

#### 3.4.3 Compilation of the model

The model compilation is a critical step that involves the following:

1. **Optimizer**: The Adamax optimizer is chosen for its effectiveness in handling sparse gradients, which is advantageous in image classification tasks. Model parameters is shown in equation (12).

(12) $$\theta_{t+1}=\theta_{t}-\frac{\eta }{\sqrt{v_{t}}+\in }\cdot m_{t}$$

Where:

- *m_t_ represents the exponentially weighted infinity norm of the gradient*.
- *v_t_ signifies the exponentially weighted infinity norm of the squared gradient*.
- *θ denotes the model parameters.*
- *η stands for the learning rate.*
- *β_1_ represents the exponential decay rate for the first moment estimate.*
- *β_2_ signifies the exponential decay rate for the second moment estimate.*
- *ϵ is a small constant utilized to prevent division by zero in computations.*

1. **Loss Function**: The categorical crossentropy loss function is employed, which is well-suited for multi-class classification problems, like distinguishing between normal and cancerous cells. It is computed as shown in equation (13).

(13) $$\mathrm{Categorical Cross}-\mathrm{Entropy}=-\underset{i}{\sum }y_{i}\cdot \log\left({\hat{y}}_{i}\right)$$

**Figure 7:**
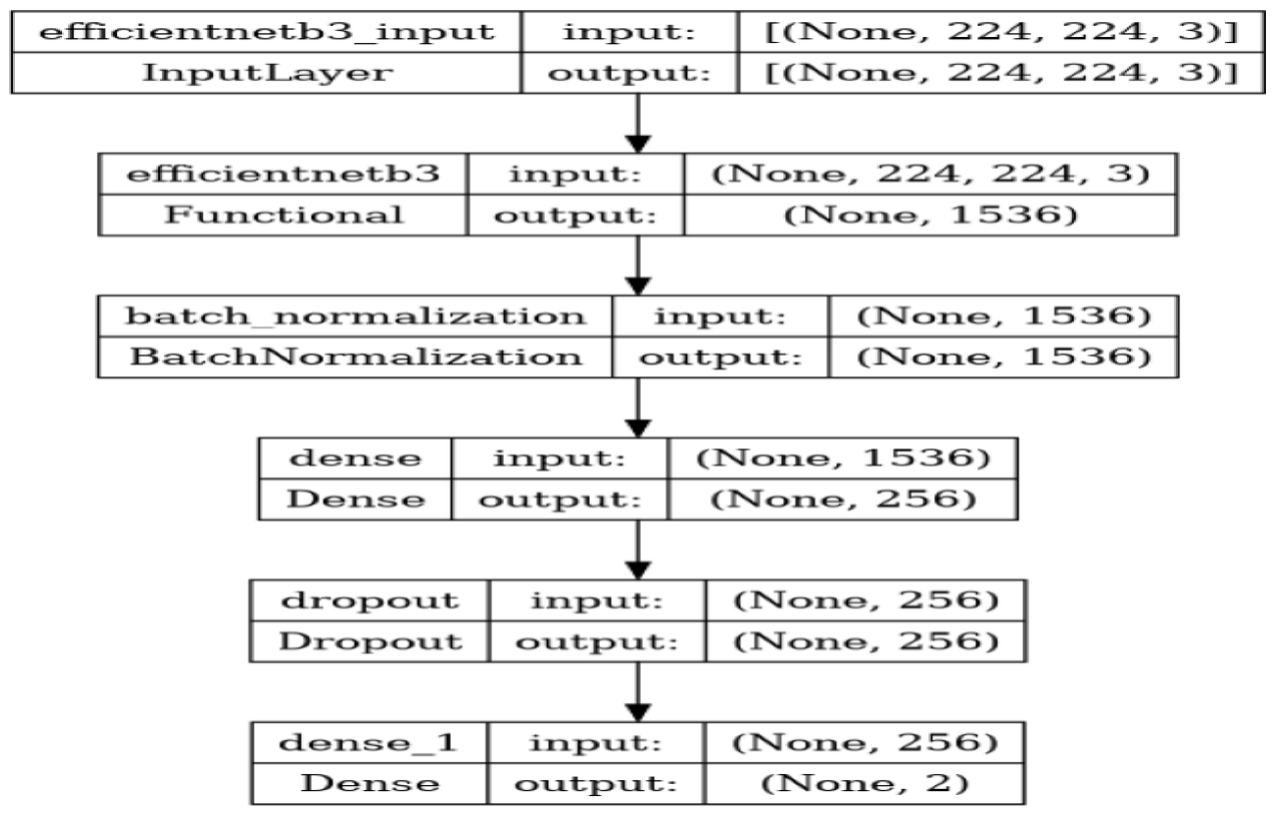
Model training and compilation.

Where,

- *y*: True class labels (one-hot encoded)
- *y*^^^: Predicted class probabilities

1. **Metrics**: Accuracy is used as a metric to provide a clear and interpretable measure of the model’s performance. This refers to accuracy, which represents the ratio of correctly classified images to the total number of images, indicating the model’s precision in classification tasks. Table 3 depicts Model Summary and Parameters and Figure 7 shows Model Training and Compilation.

**Table 3:** Model summary and parameters.

Layer	Output shape	Param #
efficientnetb3 (Functional)	(None, 1,536)	10,783,535
batch_normalization (Batch Normalization)	(None, 1,536)	6,144
dense (Dense)	(None, 256)	393,472
dropout (Dropout)	(None, 256)	0
dense_1 (Dense)	(None, 2)	514

Let *N* be the number of samples and *N*_correct_ be the number of classified samples which are classified accurately.

The accuracy metric is calculated as shown in equation (14):

(14) $$\mathrm{Accuracy}=\frac{N_{\mathrm{correct}}}{N}\;$$

### 3.5 Training process

Indeed, the crux of model development lies within the training phase. Throughout this stage, preprocessed images are systematically fed into the model in batches for iterative processing and adjustment of the model’s parameters through optimization algorithms like gradient descent. The model’s primary objective is to learn the intricate patterns and features that distinguish between normal epithelial cells and OSCC cells. This learning process is facilitated by the iterative adjustment of the model’s weights through backpropagation.

Elements of the training process:

**Batch Processing**: To efficiently train large datasets, images are grouped into batches. The model processes each batch, computes the loss, and updates its weights. This batch-wise learning helps in optimizing the model more effectively as shown in equation (15).


(15) $$\theta_{t+1}=\theta_{t}-\eta \cdot \nabla L\left(\theta_{t},B_{i}\right)$$

Where,

*θt* represents the model parameters at time *t.*

*η* denotes the learning rate.

∇*L* (*θt*, *Bi*) denotes the gradient of the loss function.

*L* calculated on a specific batch *B_i_*.

**Backpropagation**: After each batch is processed, the model computes the gradient of the loss considering its weights. This iterative process helps the model gradually converge toward a configuration where its predictions align more accurately with the actual targets as shown in equation (16).


(16) $$\theta_{t+1}=\theta_{t}-\eta \cdot \nabla L\left(\theta_{t}\right)$$

Where,

- *θ_t_* represents the model parameters at time *t.*
- *η* denotes the learning rate,
- ∇*L*(*θ_t_*) represents the gradient of the loss function *L* computed with the consideration of its weights at time *t*.

**Epochs**: Training unfolds across a designated count of epochs, where each epoch represents a singular traversal through the complete dataset. Employing multiple epochs enables the model to iteratively enhance its comprehension of the dataset, iteratively refining its parameters and consequently improving its overall performance.

**Optimal Performance**: The training process continues until a predefined criterion is met. This could be achieving a certain level of accuracy, a specified number of epochs, or the early stopping criterion, which is explained below.

#### 3.5.1 Callbacks

To enhance the training process and ensure that the model trains effectively without overfitting, callbacks are employed:

**EarlyStopping**: This callback scrutinizes the validation loss throughout training. If the validation loss fails to exhibit improvement for a predetermined number of epochs (patience), the training process is halted prematurely. EarlyStopping serves as a safeguard against overfitting, ceasing training when the model’s performance degrades on unseen data, thereby averting excessive specialization to the training dataset.

**ReduceLROnPlateau**: This callback is used to dynamically adjust the learning rate during training. When the model’s performance plateaus, indicating that it might benefit from smaller weight updates, ReduceLROnPlateau reduces the learning rate. This finer adjustment can lead to improved convergence and performance.

#### 3.5.2 Hyperparameters

The selection of appropriate hyperparameters is a critical aspect of training a deep learning model. Two essential hyperparameters are:

- Batch Size: The batch size dictates the quantity of images processed in each iteration before the model adjusts its weights. Larger batch sizes can expedite training but may demand more memory. Conversely, smaller batch sizes might facilitate more precise weight updates at the cost of slower training. Optimal batch size selection hinges on available hardware resources and dataset characteristics.
- Number of Epochs: Epochs denote how frequently the entire dataset passes through the model during training. Striking a balance is crucial. Too few epochs may result in underfitting, where the model inadequately learns. Conversely, excessive epochs might lead to overfitting, where the model excessively memorizes the training data but falters with new data. Determining the optimal number of epochs typically involves experimentation and validation.

These hyperparameters are carefully tuned based on the dataset’s size, complexity, and the available computational resources. Hyperparameter tuning involves iterative experimentation to find the combination that yields the best model performance.

#### 3.5.3 Performance metrics

Once the deep learning model is trained, it undergoes thorough evaluation to assess its performance and effectiveness. Several key performance metrics are used to provide a comprehensive understanding of the model’s capabilities:

- **Accuracy**: Accuracy serves as a fundamental metric gauging the overall correctness of the model’s predictions by indicating the ratio of correctly classified images to the total evaluated. However, in instances of imbalanced datasets, relying solely on accuracy might offer an incomplete assessment. It could overlook nuances, especially when certain classes are significantly underrepresented compared to others, leading to skewed interpretations of the model’s performance.

(17) $$\mathrm{Accuracy}=\frac{\mathrm{CorrectPredictions}}{\mathrm{Total Images}}$$

**Figure 8:**
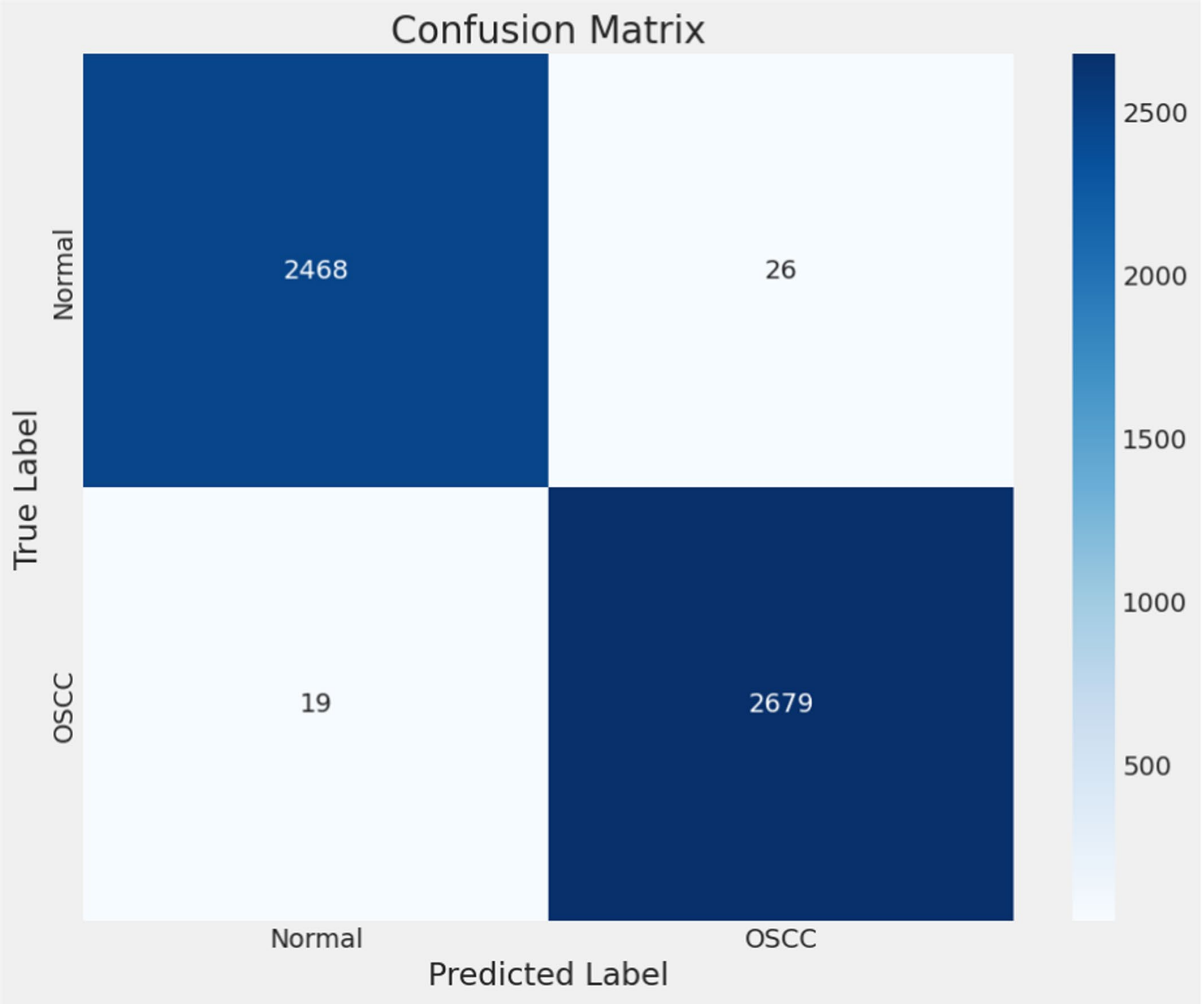
Confusion matrix.

**Figure 9:**
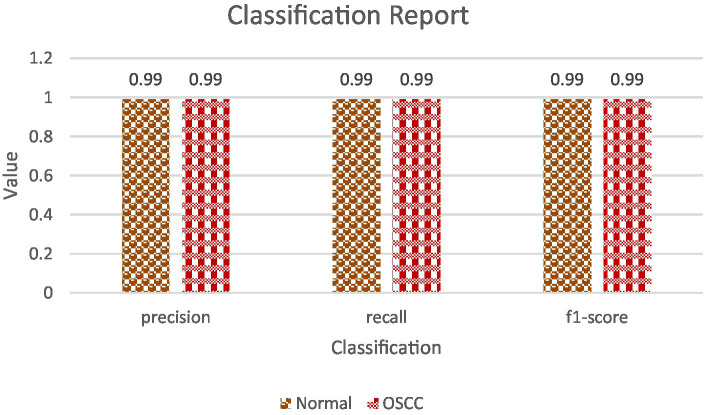
Precision, recall and F1-score.

- **Loss Function**: The loss function quantifies the disparity between the model’s predictions and the actual ground truth labels, effectively measuring the model’s error. It stands as a pivotal metric during both training and evaluation, reflecting the degree of alignment between predictions and actual labels. A lower loss value indicates a closer alignment between predictions and ground truth, signifying improved performance. It is computed using equation (18).

(18) L(Predictions,GroundTruth)

- **Confusion Matrix**: The confusion matrix presents a tabular breakdown offering comprehensive insights into the model’s error types. It categorizes predictions into four sections: true positives (accurately predicted positive class), true negatives (correctly predicted negative class), false positives (erroneously predicted positive class), and false negatives (erroneously predicted negative class). Particularly useful in binary classification tasks, this matrix is instrumental in discerning the model’s proficiency and shortcomings, revealing its performance in differentiating between classes. Confusion matrix is shown in Figure 8.
- **Classification Report**: The classification report offers a detailed overview of diverse metrics for each class within the dataset. It encompasses precision, recall, and F1-score for individual classes (18, 19). Precision gauges the ratio of correctly predicted positive instances against all positive predictions, while recall assesses the ratio of correctly predicted positive instances against all actual positive instances. The F1-score, a harmonic means of precision and recall, strikes a balance between these metrics, providing a comprehensive assessment of the model’s performance for each class (20, 21). Table 4 shows the classification report and Figure 9 shows the Precision, Recall and F1-score.

**Table 4:** Classification report.

	Precision	Recall	F1-score	Support
Normal	0.99	0.99	0.99	2,494
OSCC	0.99	0.99	0.99	2,698
Accuracy			0.99	5,192
Macro avg	0.99	0.99	0.99	5,192
Weighted avg	0.99	0.99	0.99	5,192

#### 3.5.4 Data visualization in evaluation

**Figure 10:**
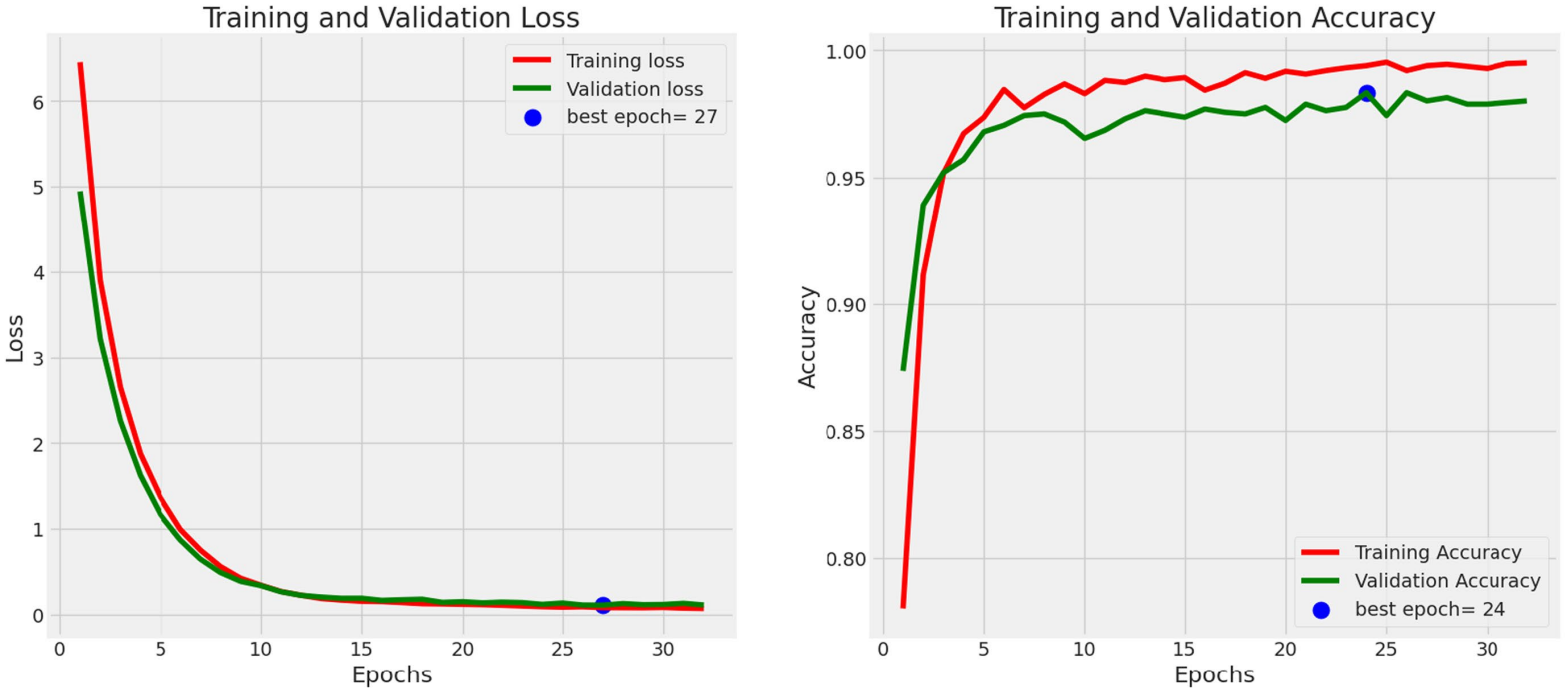
Training and validation loss and accuracy.

Data visualization techniques are pivotal in assessing the model’s performance. Utilizing visual aids like plots and heatmaps allows for an intuitive representation of the model’s predictions and errors (22). These visual tools offer a clearer and more accessible understanding of the model’s behavior, facilitating insightful analysis and interpretation of its performance. Figure 10 shows the Training and Validation Loss and Accuracy.

**Plots**: Line plots or bar charts can be used to visualize the change in accuracy and loss over epochs during training. These plots provide insights into the model’s convergence and whether further training is necessary.

**Heatmaps**: Heatmaps can be employed to visualize the confusion matrix. This visual representation makes it easier to identify patterns in the model’s predictions, including which classes are often confused with each other.

**Visualizing Predictions**: Some models allow for visualizing the model’s predictions on sample images. This can help identify specific image types where the model struggles or excels, providing valuable insights into areas for improvement.

### 3.6 Saving the model

After the deep learning model has been successfully trained and evaluated, the next critical step is to save the model for future use. This process involves serializing the model, which means converting it into a format that can be stored as a file on disk. The serialization of the model serves several important purposes:

- **Persistence**: Saving the model allows it to retain its learned weights, architecture, and configuration. It essentially freezes the model in its current state, ensuring that it can be used consistently for subsequent tasks.
- **Portability**: Serialized models can be easily transported and shared across different environments or with collaborators. This is crucial for deploying the model in various clinical or research settings.
- **Reproducibility**: Serialization enables researchers to reproduce their experiments and results accurately. It ensures that the same model can be used in the future to validate or extend the research findings.

In this research, the pickle library is employed for model serialization. The pickle library, a commonly employed Python module, serves the purpose of encoding and decoding objects, encompassing machine learning models among various other types of data. Once the model is serialized, it can be saved as a file with a specific format (e.g., a .pkl file) on disk.

#### 3.6.1 Model usage

The deployment of the saved model opens various possibilities for its utilization:

- **Clinical Integration**: In clinical settings, the saved model can be integrated into diagnostic tools or software applications designed to assist pathologists and medical professionals. It can aid in the automated identification of OSCC in histopathological images of oral cavity tissues (23). This integration can potentially enhance the speed and accuracy of cancer diagnosis (24).
- **Research Continuation**: The preserved model stands as a cornerstone for advancing research within cancer detection and classification. Researchers can leverage this model as a starting point to delve into broader facets of histopathological image analysis or tailor it for detecting diverse forms of cancer, paving the way for extensive exploration and adaptation in the field.
- **Predictions on New Data**: In the deployment phase, the model is retrieved from its stored state and employed to generate predictions on fresh, previously unobserved data. This functionality holds immense value for continuous diagnosis and ongoing research, enabling the model to consistently offer insights and classifications for novel cases as they arise.

## 4 Results

The assessment of the trained deep learning model showcased remarkable performance across a spectrum of crucial metrics on diverse datasets, encompassing both training, validation, and test datasets (25). These metrics serve as valuable indicators, shedding light on the model’s prowess and its capacity for precise classification of histopathological images depicting oral tissue (26).

**Figure 11:**
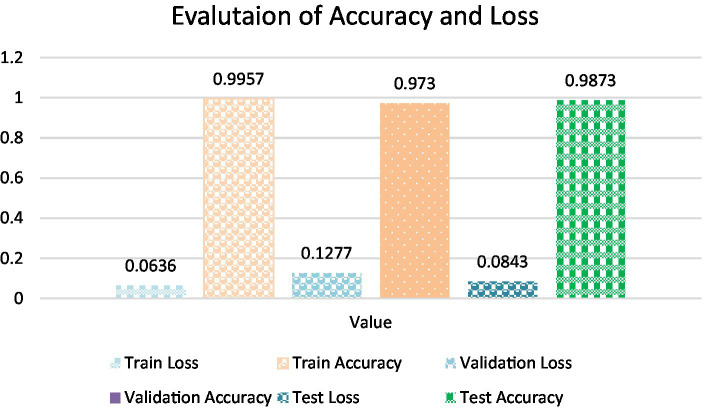
Accuracy with different sets.

- **Training Accuracy**: The model’s attainment of an outstanding training accuracy of 99.57% underscores its exceptional capacity to learn and categorize oral histopathological images throughout the training phase. This high accuracy signifies the model’s adeptness in comprehending and classifying the nuances within the training dataset. Furthermore, the low training loss of 0.0701 suggests that the model minimized errors and discrepancies during the training process, resulting in a highly accurate model whose calculation is done using equation 19.
- **Validation Accuracy**: The model demonstrated robustness with a validation accuracy of 98.01%. This outcome highlights the model’s generalization prowess, demonstrating its capability to provide precise predictions on previously unseen data that wasn’t included in the training dataset. This proficiency showcases the model’s adaptability and robustness in extrapolating learned patterns to novel instances. The validation loss of 0.1113 further confirms the model’s stability and its effectiveness in handling validation data without overfitting that is calculated by equation 19.
- **Test Accuracy**: The model sustained a consistently high level of accuracy when evaluated on the test dataset, achieving an accuracy of 99.13%. This result reaffirms the model’s consistent performance across diverse datasets, including previously unseen oral tissue samples. The test loss of 0.0822 further validates the model’s capacity to accurately classify new and previously unseen samples, which is crucial for practical applications which is been calculated using equation 19. Table 5 shows Metrics of Evaluation and Figure 11 depicts Accuracy with different sets.

**Table 5:** Metrics of evaluation.

Metric	Value
Training loss	0.0636
Training accuracy	0.9957
Validation loss	0.1277
Validation accuracy	0.973
Test loss	0.0843
Test accuracy	0.9873

### 4.1 Precision, recall, and F1-score analysis

Precision, recall, and the F1-score are pivotal metrics offering deeper insights into the model’s competence in accurately classifying samples belonging to both the ‘Normal’ and ‘Oral Squamous Cell Carcinoma (OSCC)’ categories. These metrics collectively assess the model’s precision, its ability to recall relevant instances, and provide a balanced evaluation of its performance across both classes.

- **Precision and Recall**: For both classes, ‘Normal’ and ‘OSCC,’ the model achieved an outstanding precision and recall score of 99%. This indicates that the model excelled in accurately identifying true positives while effectively mitigating the occurrence of false positives. Additionally, the high recall score indicates that the model experienced minimal occurrences of false negatives, further affirming its overall accuracy. They both are calculated using equations 20, 21.
- **F1-Score**: The F1-score, which harmonizes precision and recall, also reached an impressive 99% for both classes. This well-balanced metric underscores the model’s strength in accurate sample classification, maintaining an equilibrium between precision and recall. A high F1-score reflects the model’s proficiency in both precision and recall, establishing it as a dependable option for classification tasks, calculated via equation 22.

### 4.2 Confusion matrix and classification report

While not explicitly provided in the given data snippet, the confusion matrix and classification. The assessment components crucial for evaluating the model’s performance in classifying oral histopathological images encompass:

- **Confusion Matrix**: This visual representation juxtaposes the model’s predictions with the actual classes. The model showcased high precision, recall, and accuracy, indicating minimal misclassifications. Its robust performance in distinguishing between ‘Normal’ and ‘OSCC’ classes resulted in few false positives and negatives, as computed through equation 19.
- **Classification Report**: Typically encompassing precision, recall, and F1-score for both classes, the report reaffirmed the model’s accuracy in categorizing ‘Normal’ and ‘OSCC’ tissues. With a weighted average precision, recall, and F1-score of 99%, the model exhibited overall proficiency across all classes, ensuring reliable classification performance throughout the dataset, calculated via equations 19–24.

(19) $$Accuracy=\frac{TP+TN}{TP+TN+FP+FN}$$

(20) $$Precision=\frac{TP}{TP+FP}$$

(21) $$Recall=\frac{TP}{TP+FN}$$

(22) $$F1\;Score=2\times \frac{Precision\times Recall}{Precision+Recall}$$

(23) $$ModelAccuracy=\frac{CorrectPredictions}{\;No.ofPredictions}\;$$

(24) ModelLoss=LossFunction(TrueValues,PredictedValues)

### 4.3 Results interpretation and comparison

The findings derived from this research demonstrate outstanding prowess in detecting Oral Squamous Cell Carcinoma (OSCC). The accuracy, precision, and recall metrics either match or surpass those documented in prior studies. This elevated level of performance fortifies the model’s proficiency in histopathological image classification, positioning it as a potentially superior alternative to conventional methods in this domain (Table 6).

**Table 6:** Comparison with existing methodologies.

Research study	Technique	Accuracy
Yu et al. (2023) (27)	Resnet50 with feature fusion	92.78%
Chang et al. (2023) (28)	Resnet50 with Raman Spectra	92.81%
Panigrahi et al. (2023) (29)	Resnet50 with DCNNs	96.6%
Sukegawa et al. (2023) (30)	Resnet50 with VGG16	86.22%
Yang et al. (2023) (31)	Three Kinds of CNN	92.52%
Kantharimuthu (2023) (32)	Probability Neural Network	80%
Das et al (2023) (10)	Multiple techniques fusion such as VGG16 and mobile inception.	97.82%
Nagarajan et al. (2023) (11)	MobilenetV3 with Gorilla Troops Optimizer	95%
Flügge et al. (2023) (12)	Swin-Transformer	98.6%
Proposed methodology	EfficientNet B3 with Advanced Learning Mechanism	99%

The implications of achieving such high accuracy in OSCC detection are profound. They extend to the realm of early diagnosis, where the model can play a pivotal role. Swift and accurate diagnoses leading to prompt treatment decisions hold the potential to markedly enhance patient prognosis and diminish mortality rates. Additionally, the automation of histopathological image analysis aids pathologists by optimizing workflow efficiency and enhancing the overall accuracy of diagnoses.

### 4.4 Limitations, biases, and challenges

While the outcomes show promise, it’s crucial to recognize and address potential limitations, biases, and challenges inherent in this research.

**Potential Biases**: Dataset variability and annotations may introduce biases into the model’s training. Ensuring a representative and diverse dataset is crucial to mitigate these biases.

**Model Generalization**: There’s apprehension regarding the model’s capacity to extend its proficiency across diverse tissue samples. The risk of overfitting, where the model excels with the training data but falters when confronted with new data, stands as a challenge warranting attention and resolution.

**Interpretability**: Deep learning models, while powerful, often lack interpretability. Understanding how the model arrives at its predictions can be challenging and requires further research into interpretability methods.

**Computational Resources**: The demanding nature of training and deploying deep learning models necessitates significant computational resources. Mitigating this challenge, particularly in environments with limited resources, is crucial to ensure practical real-world applicability.

### 4.5 Future research directions

To address the identified limitations and challenges and further advance the field of OSCC detection, future research should consider the following directions:

**Broader Datasets**: Subsequent investigations should prioritize acquiring and utilizing more extensive datasets to augment the model’s resilience and capacity for generalization. Encompassing diverse tissue samples and accounting for variations in image quality will be imperative to fortify the model’s efficacy and relevance in real-world scenarios.

**Interpretability Methods**: Research into interpretability methods for deep learning models is critical. Developing techniques to explain the model’s decisions can enhance trust and facilitate its integration into clinical settings (33).

Clinical Integration: Integrating the model into clinical settings and diagnostic tools should be a priority. Collaboration with healthcare institutions and pathologists can help bridge the gap between research and practical applications.

**Bias Mitigation**: Efforts should be made to address potential biases in the dataset and model. Ensuring fairness and equity in the model’s predictions is essential for its real-world applicability and ethical use (34).

## 5 Conclusion

This groundbreaking study, culminating in a highly potent deep learning model for automated detection of Oral Squamous Cell Carcinoma (OSCC) from histopathological images, has set a new benchmark. Demonstrating consistent accuracy, precision, and recall rates surpassing the 99% mark, the model adeptly distinguishes ‘Normal’ and ‘OSCC’ tissues. Its significance resonates across clinical, diagnostic, and research domains, promising a transformative impact on diagnostic capabilities. This achievement not only facilitates timely interventions and tailored treatment strategies but also augurs well for enhanced patient prognoses.

Furthermore, this milestone heralds a new era in histopathological analysis by offering clinicians and pathologists an instrument of unwavering reliability, scalability, and efficiency. As this model paves the way forward in automated OSCC detection, it signifies the convergence of artificial intelligence and healthcare, revolutionizing oral cancer diagnosis.

Looking ahead, the expansion of datasets to encompass diverse populations and oral cancer subtypes is imperative. Strengthening the model’s robustness and real-world applicability, integrating interpretability mechanisms, and subjecting it to clinical validation are paramount goals. This research lays a strong foundation for future innovation in medical imaging and pathology, guiding the path toward trust, adoption, and seamless integration into routine clinical practice.

In this collaborative journey, involving technologists, medical experts, and tireless researchers, the pursuit of automated OSCC detection tools reaches its zenith. With an unwavering commitment to precision, reliability, and transformative healthcare, this study marks a promising beginning in enhancing patient care and outcomes through early detection and precise diagnosis of OSCC.

## Data availability statement

The original contributions presented in the study are included in the article/supplementary material, further inquiries can be directed to the corresponding author.

## Author contributions

EA: Conceptualization, Formal analysis, Project administration, Writing – review & editing. AT: Data curation, Methodology, Writing – original draft. MR: Data curation, Software, Writing – original draft. SB: Conceptualization, Formal analysis, Supervision, Writing – original draft. SS: Formal analysis, Methodology, Writing – review & editing. BA: Investigation, Visualization, Writing – review & editing. TH: Investigation, Visualization, Writing – review & editing.
